# Pseudohypoaldosteronism Type 1B and Cohen Syndrome: Novel Mutation, Unusual Combination, and Presentation

**DOI:** 10.7759/cureus.57217

**Published:** 2024-03-29

**Authors:** Yassin Alsaleh, Hussain A Al Ghadeer, Aida Aljabri, Zahra Alhashim, Moneera Mohamed, Fadi Busaleh, Fatimah A Alramadhan, Manal M Alghazal

**Affiliations:** 1 Endocrinology and Diabetes, Maternity and Children's Hospital, Al-Ahsa, SAU; 2 Pediatrics, Maternity and Children's Hospital, Al-Ahsa, SAU; 3 Endocrinology and Diabetes, Almoosa Specialist Hospital, Al-Ahsa, SAU; 4 Endocrinology and Diabetes, King Faisal General Hospital, Al-Ahsa, SAU

**Keywords:** vps13b gene, scnn1a gene, novel mutation, cohen syndrome, pseudohypoadlosteronism

## Abstract

Pseudohypoaldosteronism type 1 (PHA1) is a rare inherited disorder of resistance to aldosterone and presents with hyponatremia, hyperkalemia, and metabolic acidosis. Cohen syndrome (CS) is another rare inherited disease. Concurrent presentation with pseudohypoaldosteronism makes it so extraordinary and implies more challenges for clinicians. We report a case of a female with Cohen syndrome (novel mutation) and systemic pseudohypoaldosteronism, as well as the challenges we have encountered in the management of this patient.

## Introduction

A rare heterogeneous condition known as pseudohypoaldosteronism type 1 (PHA1) is characterized by elevated levels of renin and plasma aldosterone and neonatal salt loss in the form of hyponatremia, hyperkalemia, and metabolic acidosis. There are two main forms of PHA1 that have been identified: the autosomal dominant (AD) renal form, where the target organ defect is limited to the renal tubules, and the autosomal recessive systemic form [1-5]. Additionally described is the third form of PHA1, which is transient and frequently observed in obstructive uropathy and urinary tract infections [3]. The severe, potentially fatal salt-wasting crisis associated with the autosomal recessive form of PHA is caused by mineralocorticoid resistance in several target tissues, including the kidney, salivary gland, sweat gland, lung, and colon. The disease is caused by mutations in the epithelial sodium channel (*ENaC*) gene, which encodes a protein involved in sodium reabsorption [1-5].

Cohen syndrome (CS) is a rare, recessive inherited condition characterized by facial dysmorphism, developmental delay, and visual impairment [6-9]. The estimated global prevalence is less than 1,000 cases, as most are likely missed. This disorder affects multiple systems, including the neurological system, and is associated with ophthalmic abnormalities, dysmorphism, and obesity. There are no definitive clinical or laboratory diagnostic criteria to confirm the diagnosis of this rare disease.

The combination of CS and pseudohypoaldosteronism adds to its rarity and presents additional challenges for clinicians. We present a case of a female with CS and concurrent pseudohypoaldosteronism, as well as the challenges we encountered during her management.

## Case presentation

**Figure 1:**
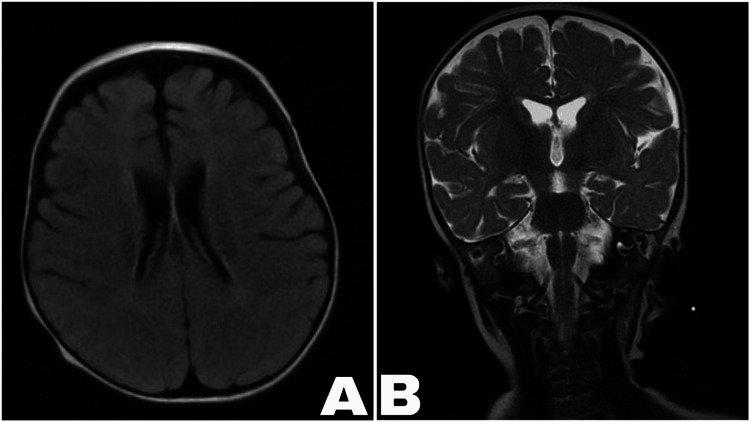
Brain MRI: (A) cross-sectional and (B) coronal images both revealed no abnormality MRI: magnetic resonance imaging

Our patient is a full-term female who was delivered via cesarean section after an uneventful pregnancy. Her weight at birth was normal. The patient was initially admitted to the neonatal intensive care unit because of birth asphyxia. During her first week of life, she developed progressive hyponatremia (115 mEq/L) and hyperkalemia (10 mEq/L), metabolic acidosis with normal anion gap, and plasma aldosterone (13,000 pmol/L), as well as plasma renin activity (PRA). Adrenocorticotropic hormone (ACTH), cortisol, and 17-hydroxyprogesterone levels were within normal ranges. To normalize the electrolytes, the patient was given a normal saline bolus, hydrocortisone, sodium bicarbonate, sodium polystyrene sulfonate resin, salbutamol, and insulin. There were no abnormalities detected by renal ultrasonography and brain magnetic resonance imaging (MRI) (Figure 1). She was admitted to the intensive care unit for a total of six weeks due to electrolyte imbalance and poor feeding. She was then diagnosed with pseudohypoaldosteronism type 1 due to elevated aldosterone, PRA, and normal adrenal and renal function. The patient was discharged home with sodium chloride, sodium polystyrene resin, and sodium bicarbonate, with regular follow-up.

The patient was readmitted to the hospital one week after discharge with a similar presentation (hyponatremia, hyperkalemia, and metabolic acidosis), necessitating resuscitation. She experienced metabolic crises and electrolyte disturbances almost every month, either spontaneously or as a result of infectious stressors, the majority of which are virally induced. A genetic study was conducted because of the difficult course of treatment, dysmorphism (thick eyebrows and eyelashes, down slanting palpebral, beaked nose, malar hypoplasia, micrognathia, protruded ears, and generalized hypotonia with depressed deep tendon reflexes with normal female genitalia), and developmental delay. Whole exome sequencing (WES) confirmed the diagnosis of pseudohypoaldosteronism type 1B (homozygous mutation in the *SCNN1A* gene) (c203_204 del variants) with Cohen syndrome (homozygous mutation in the *VPS13B* gene) (c.7522C>T variants). She is the fifth child of healthy, consanguine parents. She has an older sister (first child) who has the same dysmorphology, hypotonia, and global developmental delay, but no electrolyte imbalance (Cohen syndrome presumed). She also had an older male sibling (third child) who died at the age of one month with nearly identical symptoms, but he was not dysmorphic (probable PHA diagnosis).

Even at the age of two years, she needed to be admitted every two months. She has developed the ability to sit with help, grip objects, and transfer between hands. She recognizes her parents, has social anxiety, and uses baba and mama. Growth statistics showed failure to thrive, underweight (6.5 kg below the third centile), short suture (65 cm below the third centile), and microcephaly (41 cm below the third centile). Repeated laboratory tests revealed that her plasma aldosterone levels were normal, but her PRA levels remained elevated.

She was taking sodium chloride (14.8%) at 12.5 mEq/kg/day, sodium polystyrene sulfonate resin (0.5 g/kg/day) once daily, and sodium bicarb (2 mEq/kg/day). She continued to be on a low-potassium formula. When the family moved to another city at the age of two years and six months, she experienced acute decompensation and died.

## Discussion

Pseudohypoaldosteronism (PHA) is a rare heterogeneous condition caused by aldosterone action resistance, which leads to inadequate potassium and hydrogen secretion. Common laboratory findings include hyponatremia, hyperkalemia, metabolic acidosis, increased aldosterone, and renin. Plasma renin and aldosterone levels are significantly raised, indicating a peripheral resistance of the kidney and other tissues to mineralocorticoids. There are two types of PHA1 that may be characterized based on clinical and genetic characteristics: renal type (1A), which is inherited as autosomal dominant (AD), and systemic type with various target organ defects (1B) [1-6,10]. PHA type 1B is an autosomal recessive condition caused by mutations in the epithelial sodium channel (*ENaC*) gene (*SCNN1A*, *SCNN1B*, and *SCNN1G*) [11]. A literature review found no apparent relationship between genotype and phenotype [12,13]. Clinical manifestations are usually during the neonatal period and include severe dehydration and poor feeding, failure to thrive, hyponatremia, hyperkalemia, and metabolic acidosis. Those with the systemic type frequently have lung affections that require recurring admission due to persistent wheezing and coughing, with or without an identified bacterial airway infection. Patients often have a chronic respiratory condition similar to cystic fibrosis with a positive sweat chloride test [1-6,14]. Some individuals have experienced skin symptoms such as miliaria-like cutaneous eruptions, seborrheic dermatitis, or an eczematous rash [13,15-17]. Furthermore, aberrant lipid metabolism, cholelithiasis, and enlarged meibomian glands have been described [5,18].

PHA1 is managed with sodium supplements to correct hyponatremia, resins to reduce excessive potassium levels, and, in certain cases, sodium bicarbonate [16]. High-dose synthetic mineralocorticoids such as flurniferol, indomethacin, hydrochlorothiazide, sodium citrate, and carbenoxolone have also been tested [6,16,19]. Systemic PHA1 is a lifelong condition with little improvement over time.

Cohen syndrome is a rare autosomal recessive disorder with a wide range of clinical features, including developmental delay, microcephaly, distinctive facial dysmorphism, retinopathy, severe myopia, short stature, pubertal delay, adolescent obesity, hypermobile joints, a friendly personality, and intermittent neutropenia [7-9]. Mutations in the *VPS13B* gene, also known as *COH1*, are associated with Cohen syndrome. More than 150 mutations have been identified [20]. Kolehmainen et al. implemented criteria to help diagnose Cohen syndrome [21]. Cohen syndrome is diagnosed in a proband who has at least six of the following eight cardinal features, as well as the identification of biallelic pathogenic variants in the *VPS13B* (*COH1*) gene via molecular genetic testing: facial features of Cohen syndrome, developmental delay, microcephaly, cheerful attitude, obesity, joint hypermobility, visual abnormalities, and neutropenia. Global developmental delay is a consistent finding in all patients. Microcephaly is a characteristic feature of Cohen, but not in all patients. Hypoplasia of the cerebellar tonsils has also been reported, and MRI may reveal an enlarged corpus callosum [7]. Cohen syndrome patients typically have failure to thrive in infancy and early childhood, followed by overweight and obesity, particularly truncal obesity, which usually develops in mid-childhood and mimics the presentation of Prader-Willi syndrome. Early-onset myopia and progressive pigmentary retinopathy are two examples of visual abnormalities. Myopia usually began before the age of five and progressed over time. In a young child, retinopathy changes produce a lesion known as "bull's eye maculopathy" [7]. Other ophthalmic characteristics include night blindness, lens subluxation, astigmatism, refractive error, microphthalmia, microcornea, slow pupillary response, and iris atrophy [22]. Neutropenia is usually intermittent, with an absolute neutrophil count of less than 1.5x10^3^/uL. Cohen's endocrinopathy is associated with short stature, growth hormone deficiency, obesity, insulin resistance, diabetes mellitus, hypogonadism, and cryptorchidism [20]. Cardiac defects include valvular defects, decreased left aorta, and dilated descending aorta [7,20].

Routine monitoring of CS includes complete blood count (CBC) and absolute neutrophil count (ANC) for neutropenia. Fasting blood glucose levels should be measured on an annual basis for glucose intolerance, in addition to hemoglobin A1C, lipid profile, and blood pressure monitoring and ocular examination [20]. Life expectancy is normal; the mainstay of management is rehabilitation (occupational therapy, physiotherapy, and speech therapy). Recombinant human granulocyte colony-stimulating factor (rHG-CSF) can be used.

Back to our patient,it is well known that hyperkalemia is one of the most common findings [5,6,23] and can be difficult to treat, requiring high doses of resin up to 6 g/kg/day in one reported case [16]. In some cases, sodium polystyrene sulfonate resin was ineffective [6], and dialysis was required frequently. Our patient was extremely sensitive to resin, requiring 0.5 g/kg/day every day and occasionally every 2-3 days. Furthermore, on one occasion, the patient was kept off resin for one month while maintaining normal potassium levels. Some people prefer sodium resin to calcium resin because sodium-containing ion-exchange resins improve hyponatremia without causing hypercalcemia and hypercalciuria, which can occur in patients using calcium resins [16]. Since our patient was very sensitive, we did not see any difference.

Our case necessitated high sodium chloride maintenance doses (12.5 mEq/kg/day), whereas one reported patient required doses of up to 50 mEq/kg/day. Most cases do not require such a high dosage. The salty taste of sodium chloride makes it difficult to administer a large dose orally. So, the dosage can be divided and combined with baby formula and food [10]. A nasogastric tube/jejunostomy is sometimes required, especially if the patient is not growing properly. Regarding our patient, she was on a nasogastric tube for an on/off period, and gastrostomy was discussed with her parents. We use sodium chloride at a high concentration to reduce the amount of salt consumed. We discovered that 14.8% is an acceptable alternative to 3% for better compliance. Although fludrocortisone is used to treat congenital adrenal hyperplasia (CAH), it is not effective in systemic PHA1 [14]. Fludrocortisone was administered to our patient for three weeks but was discontinued due to two episodes of decompensation and electrolyte disturbance. We do not believe that it is related to medication, but it appears that the duration and circumstances may not be optimal.

WES confirmed pseudohypoaldosteronism type 1B by detecting a homozygous mutation in the *SCNN1A* gene (c203_204 del variants). This variant has previously been described in the literature (PMID: 8589714). However, our patient had the homozygous variant c.7522C>T p> (Gln2508*) in *VPS13B*, which resulted in stop gain and a premature stop codon. To our knowledge, this variant has not been described in the literature (HGMD 2020.4).

Neurological manifestations of Cohen syndrome existed in the form of microcephaly and hypotonia with a mixed picture of upper and lower motor neuron lesions. Examination showed depressed to absent joint reflexes, delay in sitting and standing, inability to walk independently, and delay in speech. Birth asphyxia, a stormy course of pseudohypoaldosteronism, and, of course, Cohen syndrome play a role in her global developmental delay. Our case was evaluated by an ophthalmologist, and no abnormalities were found. Echocardiography showed a small atrial septal defect (secundum) (ASD II).

The literature states that the earliest known case of neutropenia was in a three-year-old patient. Despite numerous admissions and cultures, no single bacterial organism was identified in our case. According to a review of the literature conducted by Rodrigues et al., generalized obesity is actually rare, and the link between obesity and Cohen syndrome is overstated [7]. In our case, the coexistence of PHA type 1B with frequent admission, poor feeding, and age may contribute to masking the picture and failure to thrive. Obesity in Cohen syndrome has been reported as early as two years of age.

## Conclusions

Systemic PHA1 is a life-threatening disease that should be considered as a differential diagnosis in a neonate or infant with hyponatremia, hyperkalemia, or metabolic acidosis. Patients with systemic type 1 frequently present with severe crises, necessitating prompt management to avoid catastrophic complications and mortality. The coexistence of PHA and Cohen syndrome, with overlapping and challenging presentations, necessitates a multidisciplinary team (geneticist, endocrinologist, neurologist, dietitian, etc.) to achieve optimal management and ensure adequate growth and psychomotor development, as well as genetic counseling for future pregnancies. The description of such cases may contribute to a better understanding of rare disorders, genotype-phenotype correlations, and the severity of various mutations.
